# On the Prevalence of *M. avium* Subspecies *paratuberculosis* DNA in the Blood of Healthy Individuals and Patients with Inflammatory Bowel Disease

**DOI:** 10.1371/journal.pone.0002537

**Published:** 2008-07-02

**Authors:** Ramon A. Juste, Natalia Elguezabal, Joseba M. Garrido, Andres Pavon, Maria V. Geijo, Iker Sevilla, Jose-Luis Cabriada, Angel Tejada, Francisco García-Campos, Roberto Casado, Itziar Ochotorena, Ander Izeta, Robert J. Greenstein

**Affiliations:** 1 Departamento de Producción y Sanidad Animal, Instituto Vasco de Investigación y Desarrollo Agrario (NEIKER-Tecnalia), Derio, Bizkaia, Spain; 2 Fundacion INBIOMED, San Sebastian, Spain; 3 Servicio de Aparato Digestivo, Hospital de Galdakao, Galdakao, Spain; 4 Servicio de Aparato Digestivo, Clínica Quirón Donostia, San Sebastian, Spain; 5 Servicio de Aparato Digestivo, Hospital de Txagorritxu, Vitoria-Gasteiz, Spain; 6 Department of Surgery, VA Medical Center, Bronx, New York, United States of America; The Scripps Research Institute, United States of America

## Abstract

**Background:**

Mycobacteria, such as *M. leprae* and *M. tuberculosis* infect billions of humans. However, because of appropriate immune responses and antibiotic therapy, overt mycobacterial diseases occur far less frequently. *M. avium* subspecies *paratuberculosis* (MAP) causes Johne's disease in ruminants, an affliction evocative of inflammatory bowel disease (IBD). Several agents used to treat IBD (5-ASA, methotrexate, azathioprine and its metabolite 6-MP) have recently been shown to be antiMAP antibiotics. We herein evaluate the prevalence of MAP DNA in healthy individuals and compare them with IBD patients on antiMAP antibiotics.

**Methods:**

We studied 100 healthy individuals (90 blood donors) and 246 patients with IBD. IS900 MAP DNA was identified using a nested primer PCR in the buffy coat of blood. Positive signal was confirmed as MAP by DNA sequence analysis. PCR positive results frequencies were compared according to medications used. Significance was accepted at p<0.05.

**Results:**

47% (47/100) healthy controls and 16% (40/246) IBD patients were IS900 positive (p<0.0001). MAP DNA was identified in 17% of 143 patients receiving mesalamine and 6% of 16 receiving sulfasalazine. None of the IBD patients receiving methotrexate (n = 9), 6-MP (n = 3), ciprofloxacin (n = 5) or Tacrolimus® (n = 3) had MAP DNA detectable in their blood.

**Discussion:**

We found a disquietingly large percentage of healthy individuals have MAP DNA in their blood, the significance of which remains to be determined. Counter-intuitively, the incidence of MAP DNA was significantly lower in patients with IBD. Agents with the most potent *in vitro* antiMAP activity were associated with clearance of blood MAP DNA. We posit that the use antiMAP antibiotics was responsible for the decreased prevalence of MAP DNA in patients with IBD.

## Introduction

In humans, mycobacteria often colonize without causing overt disease. In India 5% (equivalent to ∼65 million) of the population shed *M. leprae* DNA in their nasal secretions [1], although <400,000 have clinical leprosy. [2] Similarly, the number of individuals who have “latent” tuberculosis (30% of humankind) far exceeds the number with clinically evident tuberculosis. [3], [4]

*M. avium* subspecies *paratuberculosis* (MAP) causes a chronic wasting enteritis in ruminants called Johne's disease [5] that is highly evocative of Crohn's disease (CD). [6] Prevailing medical dogma [7] considers that MAP is not zoonotic. [8] Yet it is of great concern that humans worldwide are continually exposed to viable MAP. MAP has been cultured from USA chlorinated potable municipal water [9], pasteurized milk in the USA [10], and Europe [11] [12], breast milk of mothers with CD [13] and from the blood of patients with inflammatory bowel disease (IBD). [14] Intriguingly, although Koch's postulates [15] may already have been met for MAP and CD [16] they have still not been met for *M. leprae* and leprosy. [17]

We posit that the pivotal reason that MAP has not been acknowledged as a human pathogen is that, unknowingly, the medical profession has been treating MAP since 1942, when sulfasalazine was introduced. [18] Until recently, it was unrecognized that the “anti-inflammatory” 5 amino salicylic acid (5-ASA) [19] and the “immune modulators” methotrexate [20], azathioprine [21] and its metabolite 6 mercapto-purine (6-MP) [20], [21] are antiMAP antibiotics. We concluded that all antecedent studies evaluating the potential zoonotic character of MAP need to be reevaluated, as their control groups were not placebo. [19],[20]

We hypothesized that MAP may asymptomatically colonize apparently healthy individuals. We further hypothesized that the unwitting use of antiMAP antibiotics may be associated with a decrease in the incidence of MAP in patients with IBD. Accordingly, we evaluated the blood of healthy individuals and IBD patients treated with antiMAP agents for the presence of MAP DNA.

## Methods

The Ethics Committee from each institution approved this study. Every participant signed an Informed Consent form that was in compliance with all relevant national and European Union regulations. There were no therapeutic interventions or alterations in the concurrent therapy, or initiation of therapy, as a consequence of participation.

The Control group comprised 100 subjects. The majority were healthy blood donors (HBD) recruited from a regional Basque Country Blood Bank. They had met all European Union requirements for allogenic blood donation, and each denied major infections or concomitant active disease. None gave any history of gastrointestinal disease. The remainder of the Controls were healthy laboratory workers from the same region.

The IBD group consisted of 246 subjects recruited from three hospitals in the Basque Country in Northern Spain; The Quirón Donostia Clinic in Gipuzkoa (65), the Hospital de Txagorritxu in Araba (81), and the Hospital de Galdakao in Bizkaia (100) The clinical diagnosis of IBD had been predetermined by each treating physician. Patients were stratified into Crohn's disease, (CD), ulcerative colitis (UC) or Indeterminate Colitis (IC) by clinical histories and routine endoscopic, histological, and radiographic criteria. Current disease status and concurrent treatment were documented on a standardized eight item, 54 choice questionnaire that was completed by each patient with the physician's help.

### Blood sampling

Three 4 mL whole blood tubes were obtained from each subject (two sterile EDTA and one heparin-lithium Vacutainer® tubes (BD)). All blood samples were coded to conceal the patient's identity and diagnosis to laboratory workers. All samples were processed within 4 hours after extraction in a class II bio-safety cabinet.

### Nested PCR

Genomic DNA was extracted from buffy coat cells. Briefly, one volume blood was incubated with one volume 155 mM ammonium chloride for 20 minutes to lyse the red blood cells. The tube was centrifuged (10 mins. 200×g) the cell pellet washed twice with PBS, recentrifuged (10 mins. 200×g). DNA was extracted and purified (QIAamp DNA Blood Mini Kit (QIAGEN GmbH, Hilden, Germany) and stored at −20°C until amplified.

Nested PCR was used to amplify IS900 as described. [14] In brief, the first round primers (P90 and P91) amplify a 398 bp fragment and the second set (AV1 and AV2) identify a 298 bp fragment. For the first round, 10 µl of genomic DNA were added to 40 µl of PCR buffer mixture. The PCR buffer mixture consisted of 5 mM MgCl_2_, 0.2 mM dNTP, 6% DMSO, 2 µM primers and 2.5 U of Taq Polymerase (Invitrogen Ltd., Paisley, UK). In the second round, all conditions were the same except that 5 µl of the PCR product from the first round were used as DNA template. PCR cycling conditions were: 95°C for 5 min, 34 cycles of: 95°C for 1 min, 58°C for 1.5 min, 72°C for 1.5 min, with a final extension phase of 10 min at 72°C. Amplification products were separated using 2% agarose gel electrophoresis (150 volts; 50 mins). The positive MAP control was ATCC 19698 DNA and the negative controls were distilled water, identically processed with the clinical samples. A band co-migrating with the ATCC 19698 DNA at the predicted amplicon size of 298-bp was considered positive.

The identity of the amplified 298 bp amplicon was confirmed from two positive healthy controls and 2 IBD patients. Bands were excised, extracted, purified (GFX PCR DNA and Gel Band purification kit. Amersham Biosciences, Buckinghamshire, UK) and commercially sequenced (Centro Superior de Investigaciones Científicas, Madrid, Spain). The sequence identity of the final 298 bp. amplicon was compared with Genebank accession X16293 sequence for MAP IS900 using BLAST (NLM) and sequence alignment analyses.

### Contamination Avoidance Procedures

Stringent controls were adopted to minimize the possibility of contamination. These include using a Level II Bio-safety hood to process buffy coat for DNA extraction and using separate uniforms, rooms, pipettes and thermocyclers for the primary and the secondary rounds of PCR amplification.

### Statistical Analyses

Comparisons of MAP DNA in blood frequencies for overall IBD, IBD type, lesion location and type of treatment were made using the Fisher's exact test (SAS Institute Inc., Cary, NC 27513, USA). Treatments were grouped by chemical structure or presumed mechanism of action in three categories: anti-inflammatories or salycilic acid derivatives (SAD) (mesalamine and sulfasalazine), immuno-modulators (azathioprine, 6-mercaptopurine, methotrexate and Tacrolimus®) and conventional antibiotics (metronidazol and ciprofloxacin). Statistical significance was accepted at p<0.05.

## Results

Of the 100 Controls in this study, 90 were healthy human blood donors and 10 healthy laboratory workers. The majority of IBD subjects (54%; 132/246) had CD, 42% (103/246) had UC and 3% (8/246) had IC. As a group the CD were the youngest, and IC the oldest (Table 1). When anatomical location of maximal pathology was reported, a minority (5%; 6/116) of CD subjects had disease confined to the colon (Data not presented). Among subjects with UC, a minority (42%; 38/92) had ulcerative proctitis (Data not presented).

**Table 1:** Classification: Crohn's disease (CD), Ulcerative Colitis (UC), Indeterminate Colitis (IC), & Controls (Healthy Blood Donors and laboratory workers).

	CD	UC	IC	Controls
N	132	103	8	100
Age (years)	38.7	44.5	51.1	37.7
Sex male	58	47	4	57
Female	74	56	4	42
Duration of disease (years)	12.3	11.8	16.1	-
Disease Activity				
Active	22	16	2	-
Inactive	107	87	6	-
Medications*				
Salicylic acid derivatives	81	68	6	-
Anti-metabolites	41	20	1	-
Steroids	24	17	1	-
Antibiotics	11	4	1	-
Infliximab	10	2	1	-
No treatment	16	18	2	-
Anatomical Location of Maximal Disease				
Colon	6	54	2	-
Ileum	103	0	1	-
Rectum	2	38	3	-
Upper digestive tract	5	0	0	-

IBD type and sex were not recorded for 3 subjects. Affected area not reported for 30 patients. Disease activity was not recorded for 6 patients (3 treated and 3 not treated). *Multiple IBD patients were on poly-pharmaceutical therapy.

**Figure 1:**
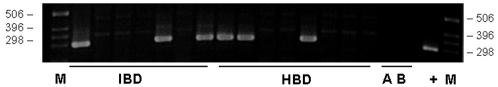
Nested PCR detection of MAP DNA from peripheral blood samples. Shown are representative samples of IBD patients and Controls. M = molecular weight marker, lane A = negative control of first round of PCR, lane B = negative control of second round of PCR, +  =  DNA from MAP strain ATCC 19698.

The PCR data (Figure 1) shows bands that co-migrate with the positive control at the predicted amplicon size of 298 bp. The sequenced DNA of the representative sample bands from each clinical subset (Controls and IBD) showed >99% identity in all cases with the Genebank accession X16293 sequence for MAP IS900 (Data not presented).

**Figure 2:**
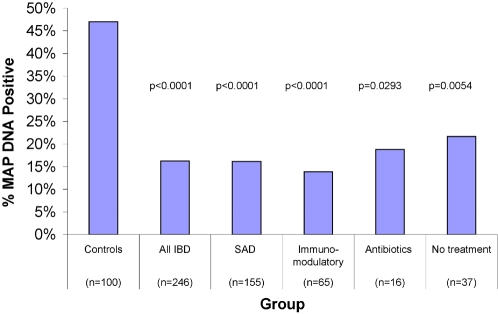
Shown is a bar graph of the % of 100 control subjects who were MAP DNA positive (first column) and all 246 patients who had IBD (second column). We next stratify IBD patients by the class of medication that they were taking. “SAD” = salicylic acid derivatives. “Anti-metabolites” were 6-MP and its precursor azathioprine, methotrexate and the “immuno-suppressive” Tacrolimus®. The conventionally accepted antibiotics used in this study were ciprofloxacin and metronidazole. The % MAP DNA positive are shown on the ordinate. The total number of 270 is greater than the number of IBD subjects (246) because some individuals were getting multiple medications and some (37) were receiving no medications at all. All IBD patients, whether combined or sub-stratified are significantly different from the non-IBD controls.

Forty seven % (47/100) of the controls [47% (42/90) blood donors and 50% (5/10) healthy laboratory workers] had a band with the predicted 298 bp amplicon (Figures 1 & 2). In contrast, 16% (40/246) IBD patients had the 298 bp amplicon (p<0.0001 compared to Controls) (Figures 1 & 2). There were no significant differences when stratified by IBD diagnosis or location of maximal pathology (Data not presented).

When comparing the prevalence of MAP DNA in each group of drugs to the Controls, conventional anti-inflammatories (p<0.0001), immuno-modulators (p<0.0001) and antibiotics (p = 0.0293) were significantly less likely to have MAP DNA in their blood. For IBD patients receiving “No medications,” MAP DNA prevalence was slightly higher (22% compared to 15% for all IBD subjects combined). However, all IBD subsets were significantly lower than the non-IBD control group. (Figure 2 Right hand columns).

Finally, within each chemical group or class of agents, we evaluated individual medications. As a *caveat*, although these data were collected prospectively, the analysis is *post hoc* and the numbers are small.

**Figure 3:**
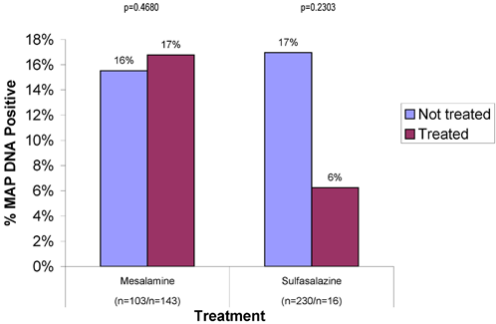
Shown is a *post hoc* analysis of the 159 IBD patients who were receiving salicylic acid derivatives. The control group comprises all IBD patients who were NOT receiving the agent identified. Mesalamine® is a proprietary name for 5-ASA. Sulfasalazine is a conjugate of 5-ASA and the antibiotic sulfapyridine. Although only 16 patients were taking sulfasalazine, the incidence of MAP DNA is significantly less than in the IBD group as a whole.

The SAD “Anti-inflammatories” were sulfasalazine and mesalamine. 17% receiving mesalamine (103 on mesalamine/143 not on mesalamine) were MAP DNA positive. Among those receiving sulfasalazine, 6% (16 on sulfasalazine/230 not on sulfasalazine) were MAP DNA positive (Figure 3).

**Figure 4:**
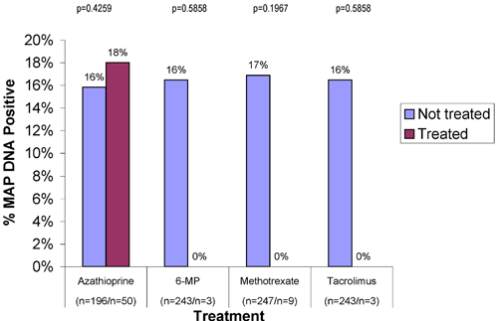
Shown is a *post hoc* analysis of the 62 IBD patients who were receiving “anti-metabolites”, agents recently shown to be potent antiMAP antibiotics. [20] [21] The control group comprises all IBD patients who were NOT receiving the agent identified. The majority were receiving the precursor of 6-MP, azathioprine. No MAP DNA is found when 6-MP, methotrexate and Tacrolimus are used.

The “Immuno-modulators” prescribed were azathioprine, its metabolite 6-MP, methotrexate and Tacrolimus®. With azathioprine 18% were MAP DNA positive, similar to controls (50 on azathioprine/196 not on azathioprine). In contrast, no MAP DNA was detected when either 6-MP (3 on 6-MP/ 243 not on 6-MP), methotrexate (9 on methotrexate; 237 not on methotrexate) or Tacrolimus (3 on Tacrolimus; 243 not on Tacrolimus) were used (Figure 4).

**Figure 5:**
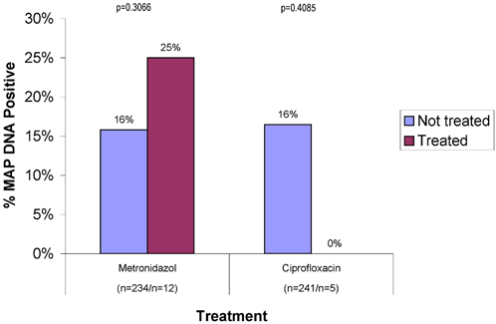
Shown is a *post hoc* analysis of the 16 IBD patients who were receiving conventional antibiotics, ciprofloxacin and metronidazole. There was no MAP DNA detected in the small number of patients taking ciprofloxacin.

The conventional antibiotics prescribed were metronidazole and ciprofloxacin. Twenty five per cent of patients receiving metronidazole were MAP DNA positive (12 on metronidazole; 234 not on metronidazole). In contrast, none of the patients on ciprofloxacin (5 on ciprofloxacin; 241 not on ciprofloxacin) were MAP DNA positive (Figure 5).

**Figure 6:**
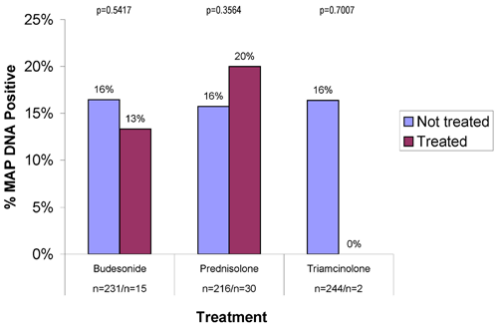
Shown are the data for the individual steroids used; prednisone, budesonide and triamcinolone. There are no significant differences noted.

**Figure 7:**
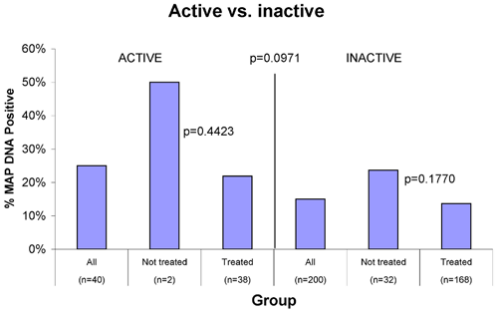
Shown is a comparison of the percentage of IBD patients who reported, at the time of phlebotomy to have “Active” (# = 40) or “Inactive disease” (# = 200). For the remaining 6 patients “Disease Activity” data were not provided on their questionnaire. In this *post hoc* analysis, with group sizes that are not comparable, there is no difference in the presence or absence of MAP DNA among the groups. Our questionnaire did NOT obtain information on medications that had been used PRIOR to the day of phlebotomy.

Steroid therapy had no effect on the presence of MAP DNA (16%) when combined (any steroid n = 44/no steroids n = 202) or individual steroids were studied (Figure 6). “Disease Activity” data were available for 98% (240/246) of IBD patients. There was no difference in the presence or absence of MAP DNA when analysed according to disease activity and/or concomitant medication usage at the time of phlebotomy (Figure 7).

## Discussion

The presence of MAP DNA does not address potential MAP viability, anymore than the presence of *M. leprae* DNA in nasal secretions [1] determines possible *M. leprae* infectivity. Nevertheless, our data shows a disquietingly high 47% of healthy individuals have MAP DNA in their blood. These data thus corroborate and extend a prior study showing 20% MAP DNA positivity in non-IBD subjects. [14] We conclude that the possible viability of MAP in blood, that may have allogenic use, should be expeditiously clarified.

“Anti-inflammatory” (5-ASA), “immuno-modulatory” agents (azathioprine its metabolite 6-MP and methotrexate) and the “immuno-suppressive” agent Tacrolimus® are used to treat IBD and multiple “inflammatory” and “autoimmune” diseases. This is despite the fact that the “mechanism of action (of 5-ASA) in the therapy of IBD is unclear” [22] and all are used simply because of empirical efficacy. In this study, we show that 5-ASA decreases the incidence of MAP DNA from the blood of patients with IBD. We additionally show that the “immuno-modulators” 6-MP and methotrexate and the “immuno-suppressive” Tacrolimus actually clear MAP DNA.

We found that all of our IBD subset groups, including those on “no active medications”, had a lower incidence of MAP DNA than the non-IBD control group (Figure 2). The most plausible explanation for the fact that IBD patients on no medication have a lower incidence of MAP DNA than the non-IBD controls is that our questionnaire did not request a history of medications that had been used by the IBD patients prior to the day of phlebotomy. Thus, those patients who were reported as being “On no medications” at the time of phlebotomy may well have been exposed to antiMAP agents in the past. (Figures 2 & 7).

Because of small numbers, our *post hoc* analyses should be considered as tentative. Nevertheless, our observations are compatible with the thesis that 5-ASA [19], azathioprine [21], 6-MP [20] [21] and methotrexate [20] are acting as antiMAP antibiotics. They also corroborate our *in vitro* data that 5-ASA [19] is a far less potent antiMAP antibiotic than are 6-MP and methotrexate. [20]

Tacrolimus [23] is an “immuno-suppressive” medication most used to prevent organ transplant rejection [24] and more recently in the therapy of IBD. [25] It is of considerable interest that Tacrolimus is from the macrolide antibiotic family of medications [23], amongst the most potent anti *M. avium* antibiotic families. [26]

Ciprofloxacin clears MAP DNA. This is an antibiotic, which has shown an activity against different strains of MAP “*in vitro.*” [27] In contrast, metronidazole an alternative antibiotic used in this study does not clear MAP DNA. Our data suggest that ciprofloxacin is a more effective antiMAP antibiotic than is metronidazole. These initial observations provide insights that should be of use when determining which agents should be evaluated when designing future, pivotal, clinical trials.

The use of the TNF alpha antagonist Infliximab® is associated with reactivation of latent tuberculosis and requires concomitant use of anti-tuberculosis prophylaxis. We find only 9% (2/13) of our patients treated with infliximab are MAP DNA positive. Our observations are therefore at variance with the 80% (4/5) culture of MAP from the blood of patients who were receiving infliximab in a prior study. [14] The most plausible explanation for these discrepant observations are the potent antiMAP agents that were concomitantly used in our patients. (Table 2). Prudence suggests that, until the potential MAP zoonosis conundrum is finally resolved, any individual being treated with TNF alpha antagonists, for any disease, should continue to receive antiMAP agents such as 5-ASA, sulfasalazine, methotrexate, 6-MP, ciprofloxacin, Tacrolimus or another macrolide antibiotics.

**Table 2:** MAP DNA in the blood of IBD patients who were receiving Infliximab® (n = 13).

	MAP DNA		Clearance Meds		Azathioprine		Steroids (Prednisolone)		No other therapy	
	%	(#/#)	%	(#/#)	%	(#/#)	%	(#/#)	%	(#/#)
Negative	85	11/13	55	6/11	36	4/11	9	1/11	9	1/11
Positive	15	2/13	0	0/2	100	2/2	50	1/2	0	0/2
**Totals**	**100**	**13/13**	**46**	**6/13**	**46**	**6/13**	**15**	**2/13**	**8**	**1/13**

“Clearance medications” are agents that, in this study, were associated with the absence of MAP DNA in the blood of IBD patient's samples. These were: methotrexate, 6-MP, ciprofloxacin, and Tacrolimus®.

When steroids are used to treat the inflammatory reaction in tuberculosis, anti-tuberculosis medications are always co-administered. [28], [29] MAP has been cultured from 60% (3/5) IBD patients receiving steroids. [14] However, our data do not show an increase in the incidence of MAP DNA when steroids are used. Again, the most reasonable explanation is the potent antiMAP agents that were co-administered in our patients being given steroids. We conclude that caution suggests that whenever steroids are used in the therapy of IBD, concomitant antiMAP agents should always be used.

If MAP zoonosis is accepted, MAP antibiotic susceptibility studies will need to be performed. However, few laboratories can successfully culture the cell wall deficient form of MAP that exists in humans [14], [30], [31], a process that may take up to 18 months. [30] Nucleic acid based methods, to more rapidly determine the presence of MAP RNA (indicating viability) [9], potential infectivity [32] [33] and antibiotic susceptibility [34] may need to be developed. Our data suggest that until such nucleic acid based methodologies are available, identifying MAP DNA prior to, and its clearance following initiation of antiMAP therapy, may provide the most readily available practicable surrogate to *in vitro* MAP antibiotic susceptibility information.
